# Analysis of clinical malaria disease patterns and trends in Vietnam 2009–2015

**DOI:** 10.1186/s12936-018-2478-z

**Published:** 2018-09-17

**Authors:** Kinley Wangdi, Sara E. Canavati, Thang Duc Ngo, Long Khanh Tran, Thu Minh Nguyen, Duong Thanh Tran, Nicholas J. Martin, Archie C. A. Clements

**Affiliations:** 1 Department of Global Health, Research School of Population Health, Australian National University, Canberra, Australia; 2 Vysnova Partners, Inc, Bethesda, MD USA; 3 National Institute of Malariology, Parasitology, and Entomology, Hanoi, Viet Nam; 4 U.S. Naval Medical Research Unit No. 2, PSA Sembawang Deptford Rd, Building 7–4, 759657, Singapore, Singapore; 5 Faculty of Health Sciences, Curtin University, Bentley, Perth, Australia

**Keywords:** Viet Nam, Malaria elimination, Bayesian, Spatial, Modelling

## Abstract

**Background:**

Viet Nam has made tremendous progress towards reducing mortality and morbidity associated with malaria in recent years. Despite the success in malaria control, there has been a recent increase in cases in some provinces. In order to understand the changing malaria dynamics in Viet Nam and measure progress towards elimination, the aim of this study was to describe and quantify spatial and temporal trends of malaria by species at district level across the country.

**Methods:**

Malaria case reports at the Viet Nam National Institute of Malariology, Parasitology, and Entomology were reviewed for the period of January 2009 to December 2015. The population of each district was obtained from the Population and Housing Census-2009. A multivariate (insecticide-treated mosquito nets [ITN], indoor residual spraying [IRS], maximum temperature), zero-inflated, Poisson regression model was developed with spatial and spatiotemporal random effects modelled using a conditional autoregressive prior structure, and with posterior parameters estimated using Bayesian Markov chain Monte Carlo simulation with Gibbs sampling. Covariates included in the models were coverage of intervention (ITN and IRS) and maximum temperature.

**Results:**

There was a total of 57,713 *Plasmodium falciparum* and 32,386 *Plasmodium vivax* cases during the study period. The ratio of *P. falciparum* to *P. vivax* decreased from 4.3 (81.0% *P. falciparum*; 11,121 cases) in 2009 to 0.8 (45.0% *P. falciparum*; 3325 cases) in 2015. Coverage of ITN was associated with decreased *P. falciparum* incidence, with a 1.1% (95% credible interval [CrI] 0.009%, 1.2%) decrease in incidence for 1% increase in the ITN coverage, but this was not the case for *P. vivax*, nor was it the case for IRS coverage. Maximum temperature was associated with increased incidence of both species, with a 4% (95% CrI 3.5%, 4.3%) and 1.6% (95% CrI 0.9%, 2.0%) increase in *P. falciparum* and *P. vivax* incidence for a temperature increase of 1 °C, respectively. Temporal trends of *P. falciparum* and *P. vivax* incidence were significantly higher than the national average in Central and Central-Southern districts.

**Conclusion:**

Interventions (ITN distribution) and environmental factors (increased temperature) were associated with incidence of *P. falciparum* and *P. vivax* during the study period. The factors reviewed were not exhaustive, however the data suggest distribution of resources can be targeted to areas and times of increased malaria transmission. Additionally, changing distribution of the two predominant malaria species in Viet Nam will require different programmatic approaches for control and elimination.

**Electronic supplementary material:**

The online version of this article (10.1186/s12936-018-2478-z) contains supplementary material, which is available to authorized users.

## Background

Despite the success in malaria control across the Greater Mekong Subregion (GMS), including Cambodia, China (Yunnan Province and Guangxi Zhuang Autonomous Region), Lao People’s Democratic Republic, Myanmar, Thailand and Viet Nam, a significant proportion of the region’s population live in malaria endemic areas, with approximately 70% of the local population at risk of contracting malaria, including 26% [1, 2] at high risk (> 1 cases per 1000 population) [3]. Some of the drivers of malaria in the GMS include favourable environmental conditions for mosquitoes competent of transmitting malaria [4, 5], frequent unchecked cross-border movement of people [6–9], movement of workers into forested border regions [6, 10, 11] and socioeconomic inequality [7, 9, 12]. In addition, the emergence of *Plasmodium falciparum* resistant to some or all anti-malarial drugs in the region has been extremely concerning [13–17]. However, over the last 15 years, malaria cases and deaths across the region have declined and all countries in the region are pursuing the goal of malaria elimination, with the last countries (including Viet Nam) aiming for elimination by 2030 [18].

Since the 1990s, Viet Nam has made tremendous progress in reducing mortality and morbidity associated with malaria [19–22]. Following a successful reduction of malaria case numbers by 97% and deaths by 99.8% between 1991 and 2014, the Viet Nam National Institute of Malariology, Parasitology, and Entomology (NIMPE) are pursuing an agenda of progressive elimination [27–30]. In 2015, a total of 68 million people were living in areas with malaria transmission [3]. In the same year, there were 15,752 confirmed malaria cases [with a near-equal proportion being caused by *P. falciparum* (54%) and *Plasmodium vivax* (46%)], and six reported deaths [3]. The decline in cases has been attributed to strengthening of the malaria control programme, increased access to health care and socio-economic improvements [20, 23, 24]. Malaria control and preventive activities that have been scaled up include early diagnosis, free treatment with artemisinin-based combination therapy (ACT), and free distribution of insecticide-treated mosquito nets (ITN) [25]. Indoor residual spraying (IRS) is carried out both as a routine preventive measure in some areas, in areas of low coverage of ITN, and also in response to outbreaks [21, 26].

Despite the success in malaria control in Viet Nam, reports in 2015 have flagged an increase in cases in some of the provinces located in Central and Central-Southern Viet Nam [27]. A number of factors are believed to be responsible for this resurgence, including: remoteness, with increasing malaria occurring in mountainous and forested areas, making control activities difficult [20], presence of the endophagic and anthropophilic vector *Anopheles dirus* [28, 29], forest-related activities [30–32], and poverty [23, 30].

Malaria transmission throughout the world is characterized by clustering of cases in transmission “hotspots”, driven by climatic, ecological and human factors. Malaria has the potential to spread from clusters into neighbouring regions and countries if interventions in the hotspot areas are not sustained. Focused interventions in areas with higher incidence of malaria are likely to have a greater impact than uniform resource allocation [33]. The recent increase in malaria cases in some areas poses a significant threat of reintroduction into areas where control has been successful, potentially derailing the NIMPE’s national goal of malaria elimination in Viet Nam.

Spatial epidemiological tools (including Geographical Information Systems [GIS] and spatial analytic methods) can be used to estimate and quantify patterns of malaria risk as well as identify environmental correlates of risk [8, 34–39]. Identification of clusters of malaria cases can help in the delineation of problem areas, which can lead to further investigation to identify possible causes of higher incidence of malaria in particular areas [40].

The objectives of this study were to identify malaria clusters in the country at the district level by species, assess correlations between environmental conditions and preventive measures including ITN and IRS coverage at the district with malaria cases, and to identify areas of the country with significantly higher trends in malaria incidence than the country as a whole, thereby identifying areas for further investigation and focussed interventions to prevent malaria resurgence.

## Methods

### Study site

**Fig. 1:**
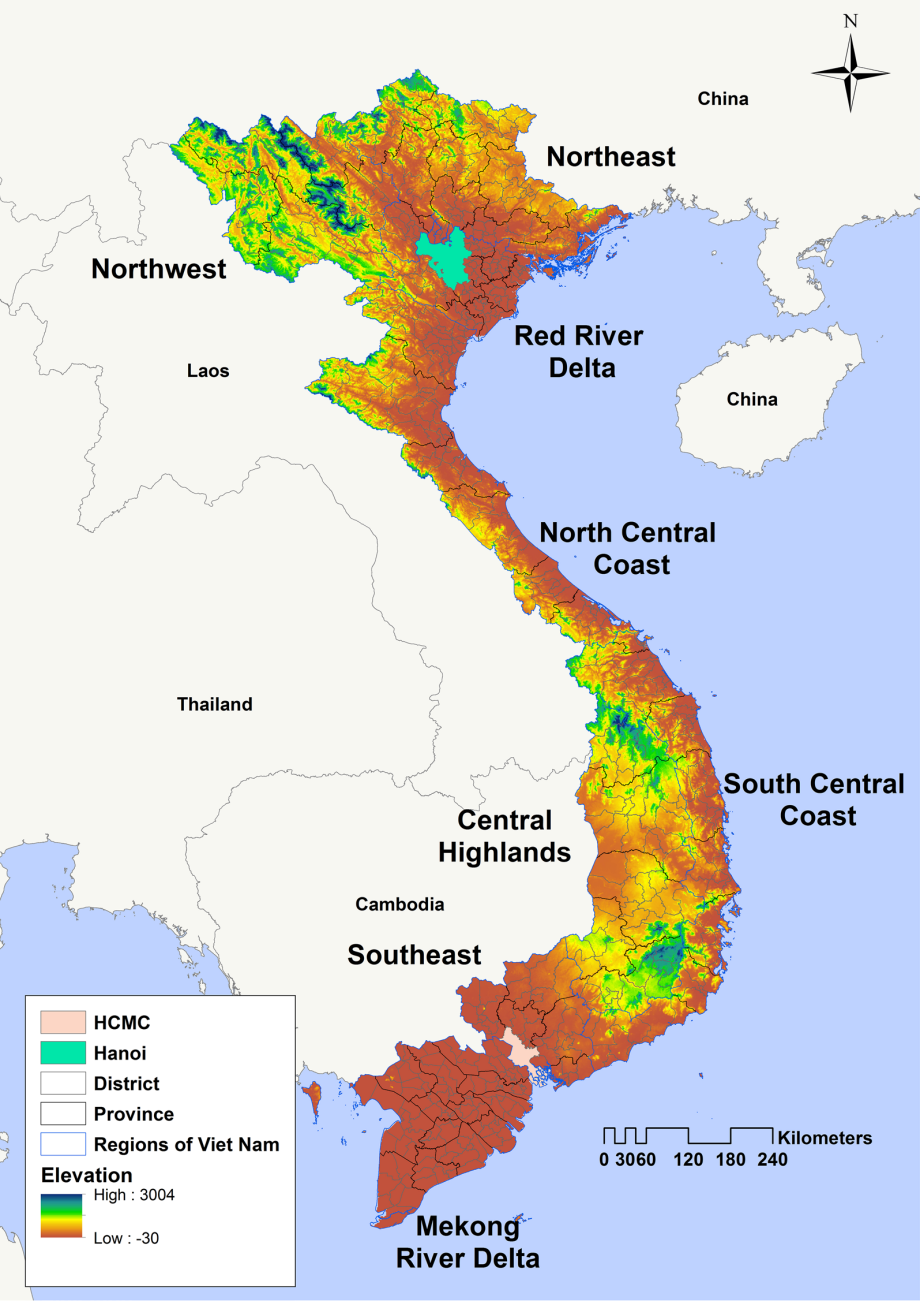
Map of Vietnam with administrative divisions and neighbouring countries

Viet Nam is located in the GMS, bordering China in the north, Cambodia and Laos to the west and the South China Sea to the East and South. It is elongated in a north–south direction and has wide variability in elevation from sea level (coastal areas) to > 3000 m near Sa Pa (Fanispan at 3142 m) in the central and northern highland areas (Fig. 1). The country is divided administratively into eight regions, 64 provinces, 702 districts and 11,100 communes.

### Data source

Reported malaria cases stratified by species and district were obtained from the NIMPE from January 2009 to December 2015. Population estimates for each year were extrapolated from the Population and Housing Census 2009 by applying an annual population growth rate of 1.06% [41].

Long-term average annual and seasonal temperature and altitude variables were created using data retrieved from WorldClim at 1 km spatial resolution [42]. These layers were produced by using a thin-plate smoothing spline algorithm to interpolate data collected from global weather station sources between 1950 and 2000. Spatial units for this analysis were districts. An electronic map of district boundaries in shapefile format was obtained from the Global Administrative Areas database (http://www.gadm.org/country). Administrative boundaries in Viet Nam have changed over time and in order to match the downloaded district boundary map from the Global Administrative Areas website, malaria data were reconciled into 678 districts.

### Crude standardized morbidity ratios

An initial descriptive analysis of malaria incidence was conducted. Crude standardized morbidity ratios (SMRs) for each district were calculated using the following formula:

$$Y_{i} = \frac{{O_{i} }}{{E_{i} }}$$

where *Y* is the overall SMR in district *i, O* is the total number of observed malaria cases in the district and *E* is the expected number of malaria cases in the district across the study period. The expected number was calculated by multiplying the national incidence by the average population for each district over the study period.

### Independent variable selection

A preliminary Poisson regression was undertaken to select covariates, with the dependent variable being number of malaria cases and independent variables being control measure and climatic variables. Climatic variables (maximum, minimum and mean temperature, precipitation, and altitude) without a lag, and with 1, 2 and 3-month lag times, and control variables were entered into univariate models. Maximum temperature without a lag and altitude had the lowest values of the Akaike’s information criterion (AIC) and Bayesian information criterion (BIC) (Additional file 1: Appendices S1, S2, S3 and S4). Maximum temperature and altitude were highly co-linear when tested for co-linearity. The model improved significantly when altitude was dropped from the model, therefore, in the final model the independent variables were control measures (ITN and IRS) and maximum temperature.

### Exploration of seasonal patterns and temporal trends

The average monthly numbers of malaria cases by *Plasmodium* species were calculated for the full time-series (January 2009–December 2015). These were plotted to show temporal patterns in malaria and climate variables. The time series of malaria incidence was decomposed using seasonal-trend decomposition based on locally weighted regression to show: the seasonal pattern, the temporal trend and the residual variability. The time series data, the seasonal component, the trend component and the remainder component are denoted by *Y*_*t*_, S_*t*_*, T*_*t*_*, R*_*t*_ respectively, for month *t* = 1 to N, and:

$$Y_{t} = S_{t} + T_{t} + R_{t}$$

The parameter setting “periodic” was used for the seasonal extraction, and all other parameters were by default. In the study, logarithmic transformations were used for the time series data [34, 43].

### Spatio-temporal model

The spatio-temporal models were developed using data from 2009 to 2014 due to difference in the number of districts between 2009 and 2014 (578) and 2015 (678). In this study, Zero-inflated Poisson (ZIP) regression was selected due to the large number of zero cases in the study. Of the 41,616 observations in the data sets, there were 29,915 (71.9%) zero counts for *P. falciparum* and 34,139 (82.0%) zero counts for *P. vivax*. ZIP models were developed in the Bayesian statistical software WinBUGS version 1.4 (Medical Research Council, Cambridge, UK and Imperial College London, UK) for *P. falciparum* and *P. vivax*. Alternative models were tested for each species, including models that include climatic variables (temperature), IRS, and ITN coverage as explanatory variables, and spatially structured and unstructured random effects. Models also included a spatiotemporal random effect that estimated spatial variability in district temporal trends. The best model based on the lowest deviation information criterion (DIC) was selected as the final explanatory model for each species.

The most comprehensive model, which had as an outcome the observed counts of malaria, *Y*, for *i*th district (*i *= 1…578) in the *j*th month (January 2005–December 2014) was structured as follows:

$$P(Y_{ij} = y_{ij} ) = \left\{ {\begin{array}{*{20}l} {\omega + 1 \left( {1 - \omega } \right)e^{ - \mu } , \quad y_{ij} = 0} \\ {\left( {1 - \omega } \right)e^{ - \mu } \mu_{ij}^{{y_{ij} }} /y_{ij} , \quad y_{ij} > 0;} \\ \end{array} } \right.$$

$$Y_{ij} \sim {\text{ Poisson }}\left( {\mu_{ij} } \right)$$

$${ \log }\left( {\mu_{ij} } \right) \, = { \log }\left( {{\text{E}}_{ij} } \right) \, + \theta_{ij}$$

$$\theta_{ij} = \alpha + \beta_{1} \times {\text{ ITN}}_{ij} + \beta_{2} \times {\text{ IRS}}_{ij} + \, \beta_{3} \times {\text{ Tmax}}_{ij} + \, \beta_{4} \times {\text{ trend}}_{j} + {\text{ u}}_{i} + {\text{ s}}_{i} + {\text{ w}}_{ij}$$

where E_*ij*_ is the expected number of cases (acting as an offset to control for population size) in district *i*, month *j*, and θ_*ij*_ is the mean log relative risk (RR); α is the intercept, and *β*_*1*_*, β*_*2*_*, β*_*3*_, and *β*_*4*_ the coefficients for proportion of population covered by ITNs and IRS, maximum temperature, and overall temporal trend of malaria risk respectively; u_*i*_ is the unstructured random effect (assumed to have a mean of zero and variance σ_u_^2^); s_*i*_ is the spatially structured random effect (assumed to have a mean of zero and variance σ_s_^2^); and w_*ij*_ is the spatiotemporal random effect (assumed to have a mean of zero and variance of σ_w_^2^).

A conditional autoregressive (CAR) prior structure was used to model the spatially structured random effect and the spatiotemporal random effect (permitting smoothing of the district-level temporal trends). Spatial relationships between the districts were determined using an adjacency weights matrix, whereby if two districts shared a border, a weight of 1 was assigned, while if they did not, a weight of 0 was assigned. A flat prior distribution was specified for the intercept, whereas a normal prior distribution was used for the coefficients. The priors for the precision of unstructured and spatially structured random effects and the spatiotemporal random effects, were specified using non-informative gamma distributions with shape and scale parameters equal to 0.01.

An initial burn-in of 10,000 iterations was run and these iterations were discarded. Subsequent blocks of 20,000 iterations were run and examined for convergence. Convergence was assessed by visual inspection of posterior density and history plots and occurred at approximately 100,000 iterations for each model. Ten thousand values from the posterior distributions of each model parameter were stored and summarized for the analysis (posterior mean and 95% credible intervals).

In all analyses, an α-level of 0.05 was adopted to indicate statistical significance (as indicated by 95% credible intervals (95% CrI) for relative risks (RR) that excluded 1). ArcMap software (ESRI, Redlands, CA) was used to generate maps of the posterior means of the unstructured and structured random effects and the spatiotemporal random effects obtained from the three models.

## Results

### Descriptive analysis

**Fig. 2:**
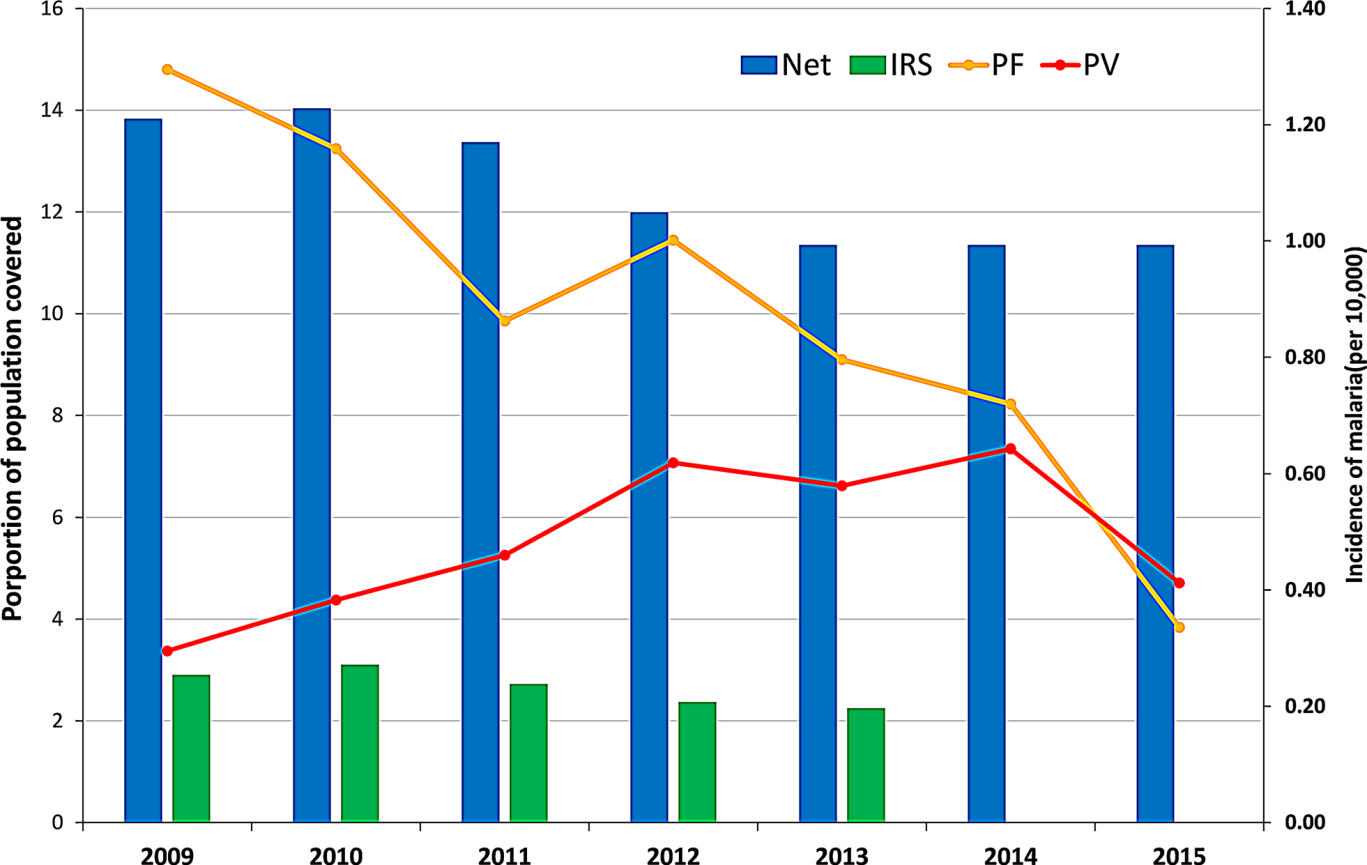
Malaria incidence with proportion of population covered by two different interventions, 2009–2015. ITN: insecticide-treated mosquito nets; IRS: insecticide residual spraying; PF: *Plasmodium falciparum;* PV: *Plasmodium vivax*

**Fig. 3:**
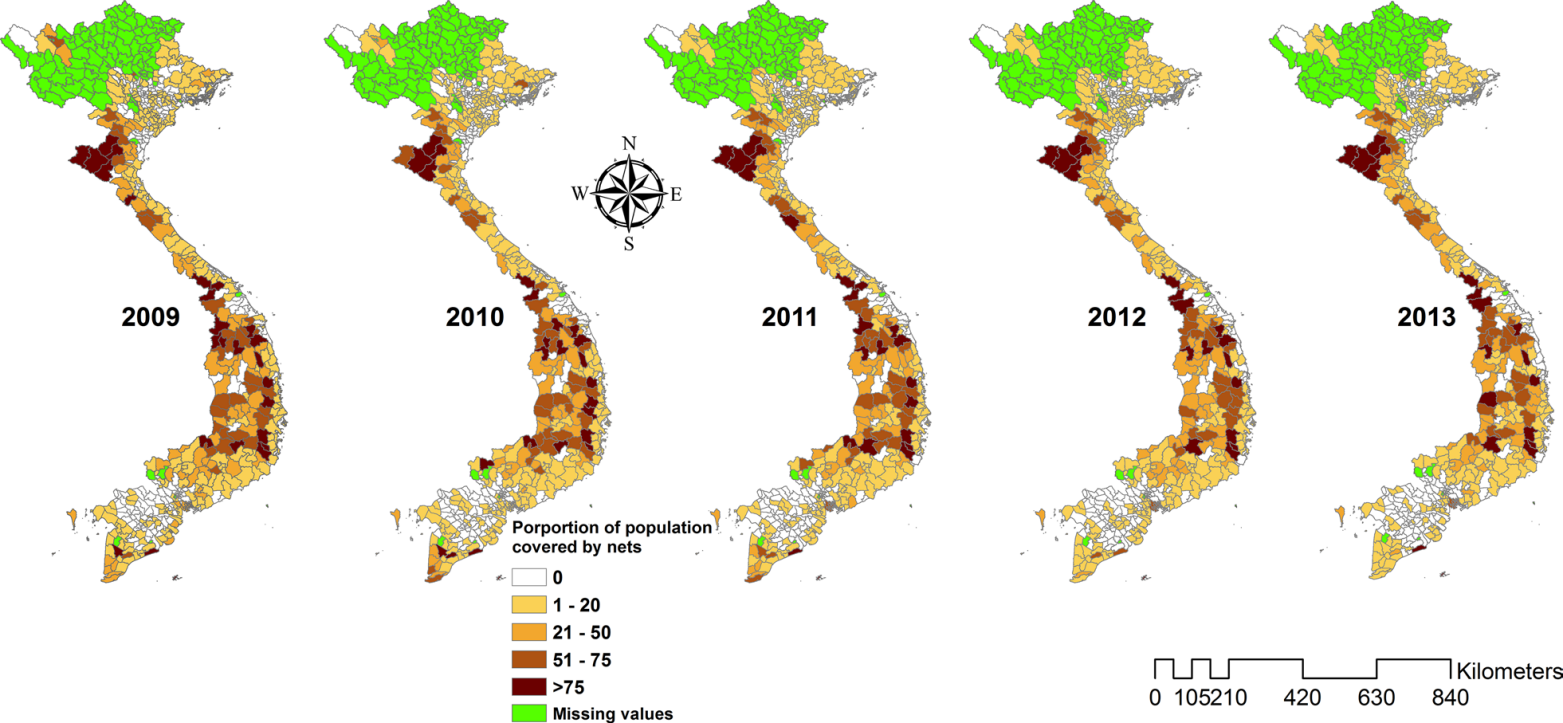
Maps showing the proportion of population protected by insecticide treated nets in districts of Viet Nam from 2009 to 2013

**Fig. 4:**
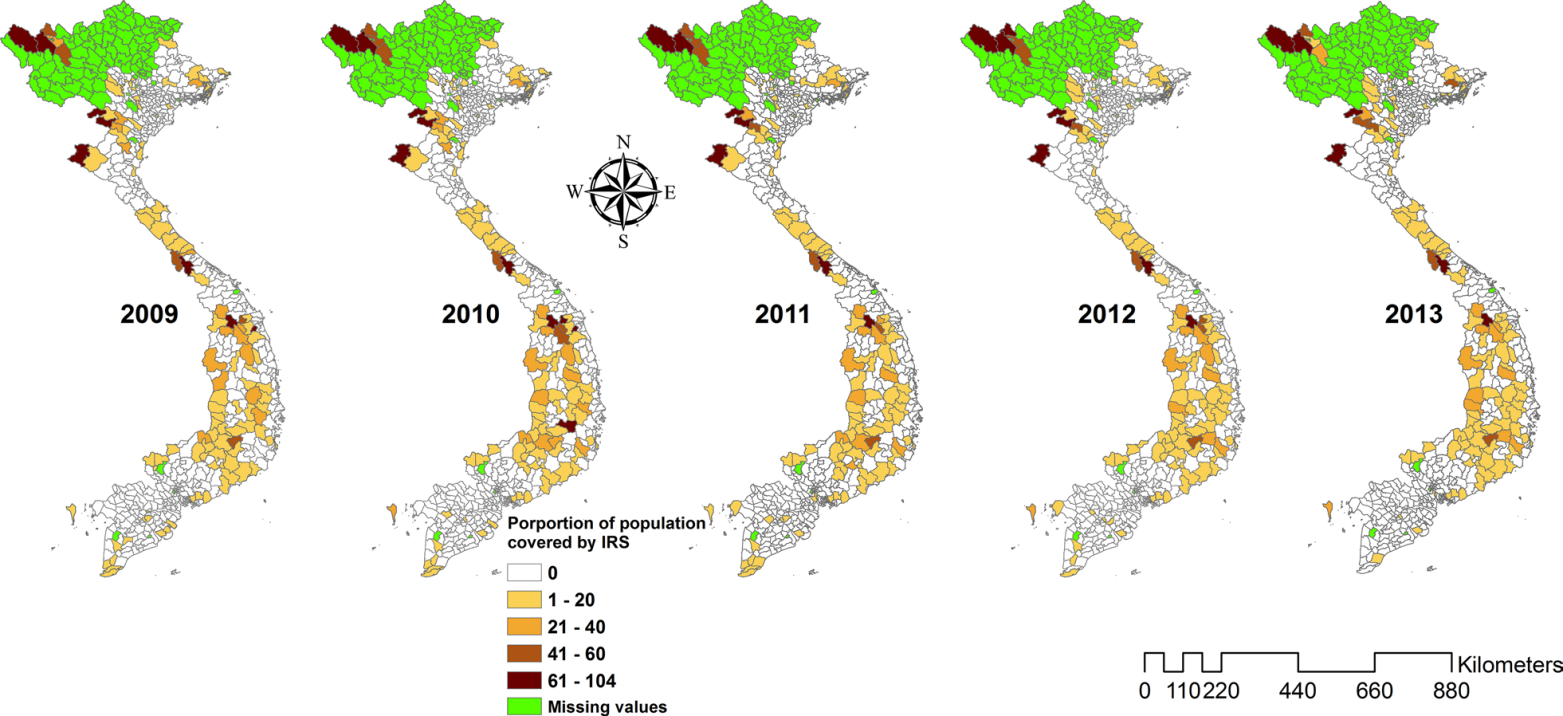
Maps showing the proportion of population protected by indoor residual spraying in districts of Viet Nam from 2009 to 2013

There was a total of 57,713 *P. falciparum* and 32,386 *P. vivax* cases during the study period. The ratio of *P. falciparum* to *P. vivax* decreased from 4.3 (81.0% *P. falciparum*; 11,121 cases) in 2009 to 0.8 (45.0% *P. falciparum*; 3325 cases) in 2015. The incidence rate of *P. falciparum* declined from 1.29 per 10,000 in 2009 to 0.34 per 10,000 in 2015, while the incidence rate of *P. vivax* varied throughout the same time with 0.30 and 0.41 per 10,000 (range 0.30 to 0.64 per 10,000) in 2009 and 2015, respectively (Table 1). The proportion of the population covered by ITNs and IRS declined slightly from a high in 2010 (14% ITNs and 3% IRS coverage at the national level) through the study period (Figs. 2, 3 and 4). However, coverage of ITNs ranged from 22.6% to 67.6% after stratification of districts that received ITNs as opposed to all the districts (Additional file 1: Appendices S5 and S6).

**Table 1:** Malaria incidence during the study period (2009–2015)

Year	*Plasmodium falciparum*			*Plasmodium vivax*		
	Cases	Proportion by year	Incidence per 10,000	Cases	Proportion by year	Incidence per 10,000
2009	11,121	0.81	1.29	2536	0.19	0.30
2010	10,745	0.75	1.16	3551	0.25	0.38
2011	8093	0.65	0.86	4319	0.35	0.46
2012	9628	0.62	1.00	5948	0.38	0.62
2013	7691	0.58	0.80	5601	0.42	0.58
2014	7110	0.53	0.72	6350	0.47	0.64
2015	3325	0.45	0.34	4081	0.55	0.41
Total	57,715			32,386

**Fig. 5:**
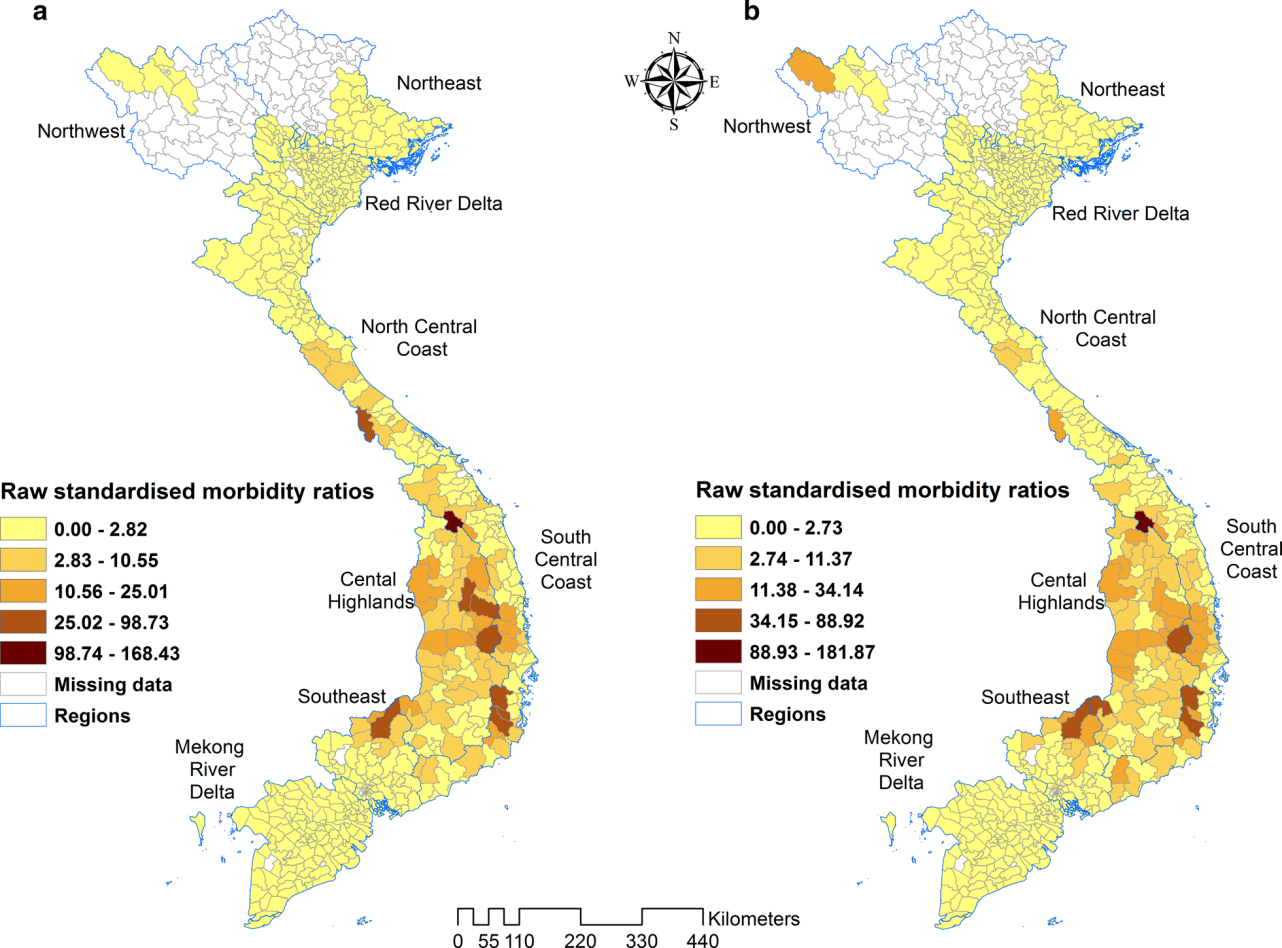
Raw standardised morbidity ratios of **a** *Plasmodium falciparum* and **b** *Plasmodium vivax* by districts in Viet Nam from 2009 to 2014

A general pattern observed in the map of SMR of *P. falciparum* was high risk in the Southeast, Central Highlands and South Central Coast regions of the country and low risk in the northern parts of Viet Nam. The distribution of *P. vivax* SMRs was similar to *P. falciparum*, but with further extension of high-risk areas to the Northwest (Fig. 5).

### Time-series decompositions

**Fig. 6:**
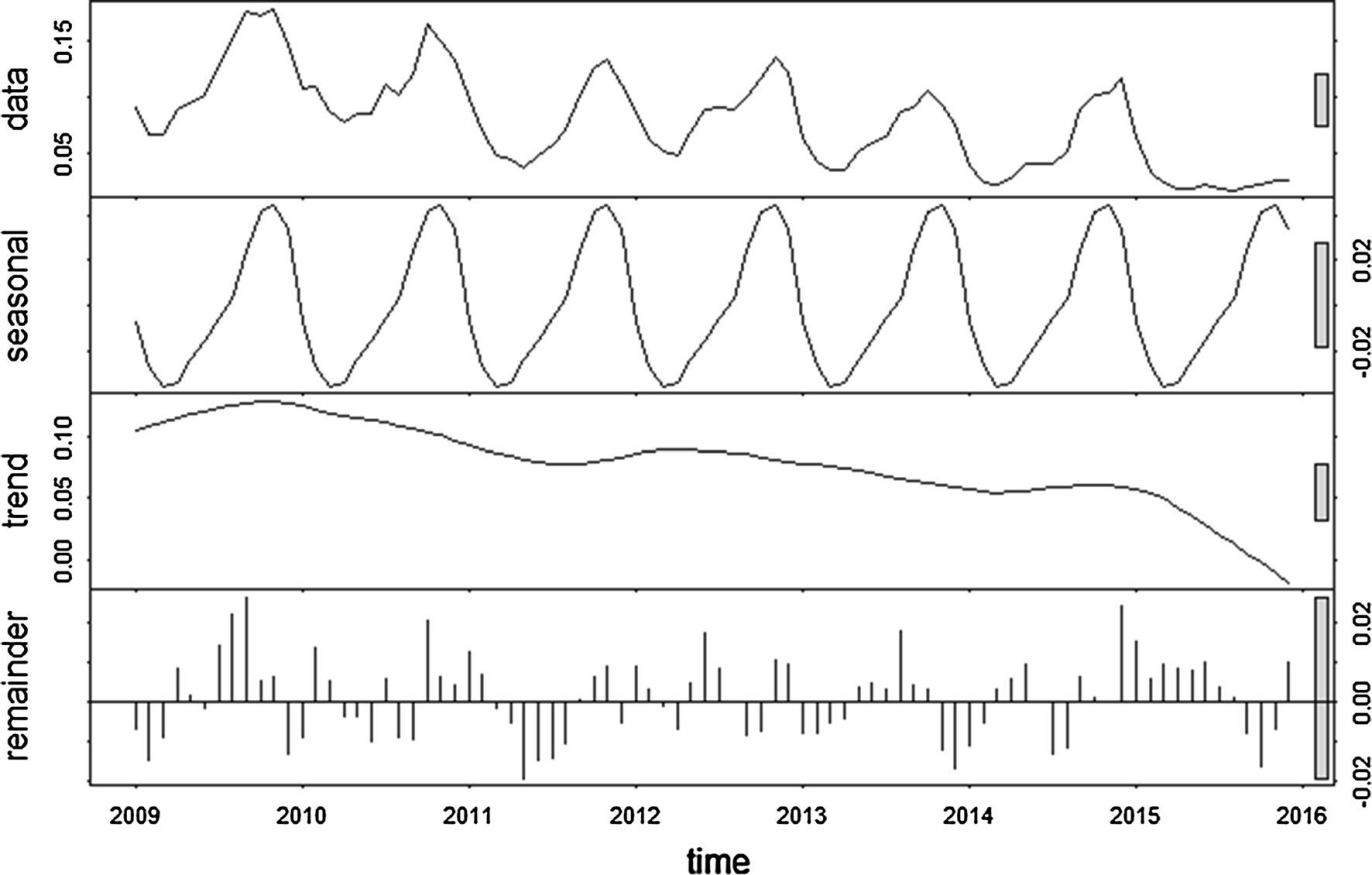
Decomposed monthly *Plasmodium falciparum* incidence per 10,000 population during the study period, 2009–2015. The top layer shows the original time series. The other layers show the decomposed components, denoting the seasonal component, long-term trend component and remainder component, respectively

**Fig. 7:**
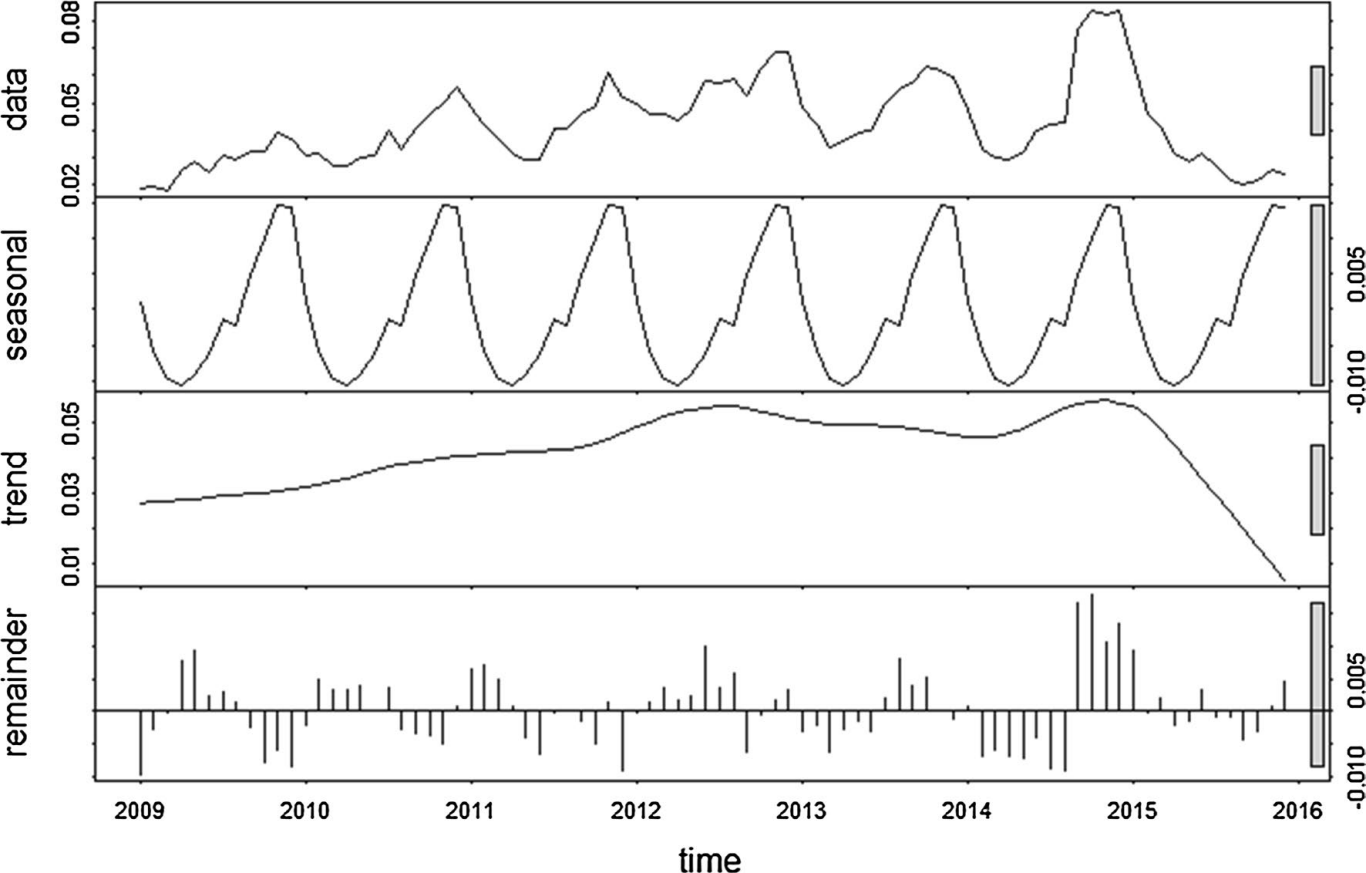
Decomposed monthly *Plasmodium vivax* incidence per 10,000 population during the study period, 2005–2015. The top layer shows the original time series. The other layers show the decomposed components, denoting the seasonal component, long-term trend component and remainder component, respectively

The time-series decompositions over the study period showed a similar seasonal patterns for *P. falciparum* and *P. vivax*. However, only one peak was seen for *P. falciparum*, whereas for *P. vivax* there were two seasonal peaks: a smaller peak in the middle of the year, followed by a larger one towards the end of the year, with the smaller peak occurring on the shoulder of the larger one. The inter-annual pattern showed a general decline in incidence of *P. falciparum*, but a general increase in incidence of *P. vivax* until 2014 (with a drop in 2015) (Figs. 6, 7).

### Spatio-temporal model

Table 2 describes the three models evaluated for spatio-temporal modelling, Model III, containing the unstructured and the spatially structured random effect, and the spatiotemporal random effect had the best fit among all the models examined for both *P. falciparum* and *P. vivax*, as indicated by the lowest DIC (Table 2). For *P. falciparum*, there was an estimated decrease of 1.1% (95% CrI 0.01%, 1.2%) in risk for each 1% increase in ITN coverage, while IRS did not have any protective effect with an increase in risk of 0.9% (95% CrI 0.7%, 1.0%) for a 1% increase in population IRS coverage. Risk increased by 3.9% (95% CrI 3.5%, 4.3%) for each 1 °C increase of maximum temperature. For *P. vivax*, coverages of preventive measures (ITNs and IRS) were associated with increased risks of 1.0% (95% CrI 0.9%, 1.4%) and 0.5% (95% CrI 0.3%, 0.7%) per 1% increase in coverage of ITN and IRS, respectively. For each 1 °C increase in maximum temperature, *P. vivax* risk was found to increase by 1.6% (95% CrI 0.9%, 2.0%).

**Table 2:** Regression coefficients and 95% CrI from Bayesian spatial and non-spatial models of *Plasmodium falciparum* and *P. vivax* cases reported by month and district, Viet Nam, 2005–2014

	*Plasmodium falciparum* RR (95% CrI)	*Plasmodium vivax* RR (95% CrI)
Model I		
Intercept^a^	− 1.67 (− 1.94, − 1.35)	− 1.93 (− 2.24, − 1.72)
Treated net coverage^b^	0.99 (0.988, 0.99)	1.012 (1.01, 1.013)
IRS^b^	1.009 (1.007, 1.11)	1.005 (1.004, 1.007)
Temp max (degree celsius)	1.039 (1.035, 1.044)	1.01 (1.006, 1.014)
Mean monthly trend	1.003 (1.000, 1.005)	0.99 (0.987, 0.992)
Probability of extra zero	0.15 (0.15, 0.16)	0.20 (0.19, 0.21)
Heterogeneity^a^		
Unstructured	0.13 (0.11, 0.14)	0.17 (0.14, 0.19)
Structured (spatial)		
Structured (trend)	0.96 (0.80, 1.13)	1.14 (0.92, 1.39)
DIC	118700	53474.2
Model II		
Intercept^a^	− 1.69 (− 1.75, − 1.63)	− 1.88 (− 1.97 − 1.80)
ITN coverage^b^	0.99 (0.987, 0.992)	0.92 (0.91 0.92)
IRS^b^	1.039 (1.035, 1.044)	1.04 (1.03 1.05)
Temp max (degree celsius)	1.008 (1.005, 1.01)	1.009 (1.005 1.01)
Mean monthly trend	1.003 (1.000, 1.005)	1.003 (1.002, 1.004)
Probability of extra zero	0.16 (0.14, 0.17)	0.20 (0.18 0.22)
Heterogeneity^a^		
Unstructured		0.19 (0.16 0.23)
Structured (spatial)	0.93 (0.15, 0.20)	1.13 (0.91 1.39)
Structured (trend)	0.18 (0.77, 1.10)	
DIC	119226	53527
Model III^c^		
Intercept^a^	− 1.78 (− 1.90, − 1.64)	− 1.88 (− 1.98, − 1.79)
ITN coverage^b^	0.989 (0.988, 0.991)	1.01 (1.009, 1.014)
IRS^b^	1.009 (1.007, 1.01)	1.005 (1.003, 1.007)
Temp max (degree censuis)	1.04 (1.035, 1.043)	1.016 (1.009, 1.02)
Mean monthly trend	1.003 (1.000, 1.005)	0.988 (0.985, 0.99)
Probability of extra zero	0.15 (0.14, 0.16)	0.20 (0.18, 0.22)
Heterogeneity^a^		
Unstructured	0.88 (0.62, 1.32)	2.52 (1.52, 4.32)
Structured (spatial)	0.38 (0.26, 0.54)	0.29 (0.21, 0.38)
Structured (trend)	0.94 (0.78, 1.12)	1.14 (0.91, 1.39)
DIC	118695	53436.7

*CrI* credible interval, *ITN* insecticide-treated mosquito net, *IRS* indoor residual spraying, *DIC* deviation information criteria

^a^ Co-efficient

^b^ Proportion of population by preventive measures

^c^ Best fit model

**Fig. 8:**
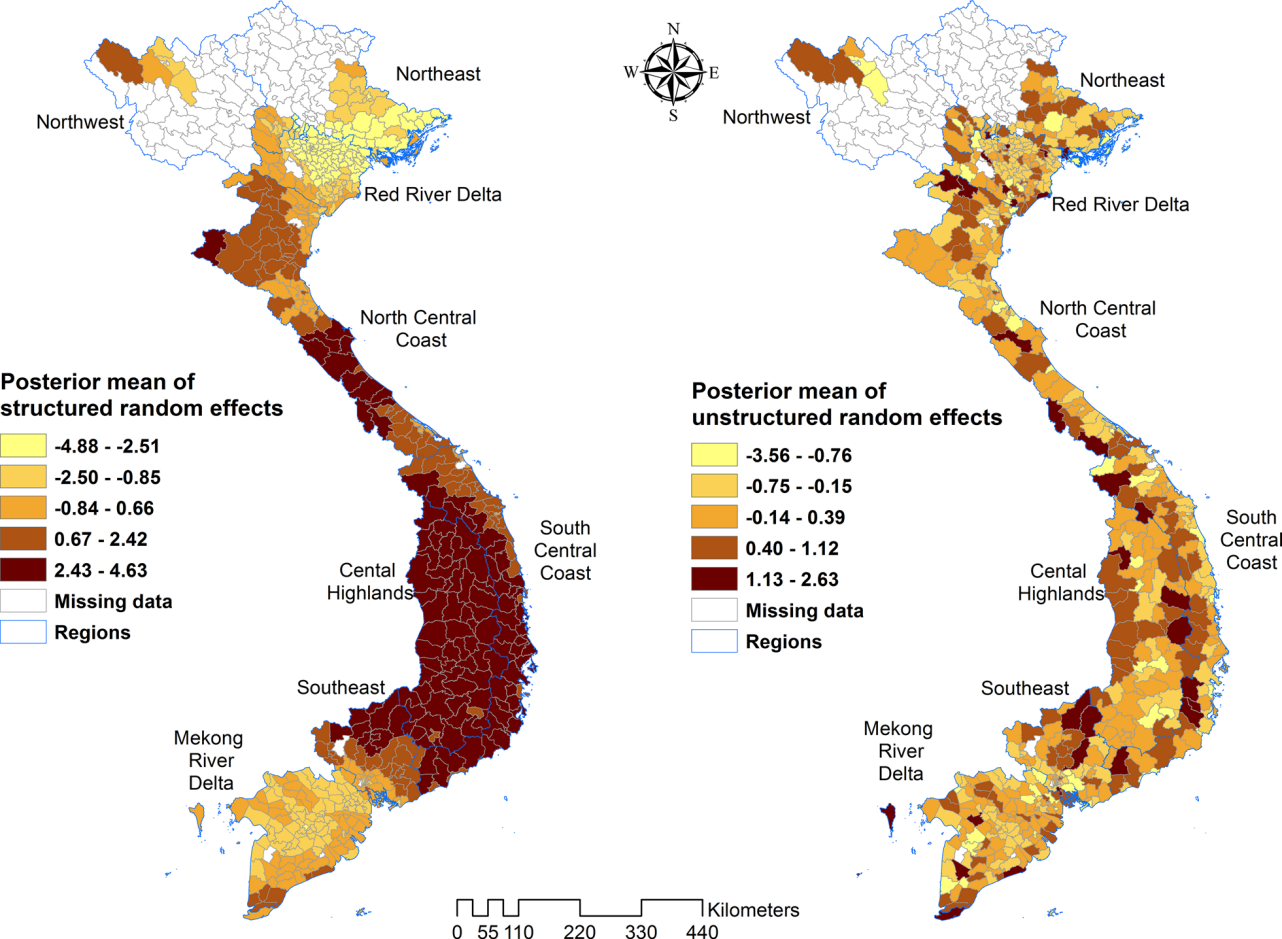
Spatial distribution of the posterior means of structured and unstructured random effects for *Plasmodium falciparum* in Model III

**Fig. 9:**
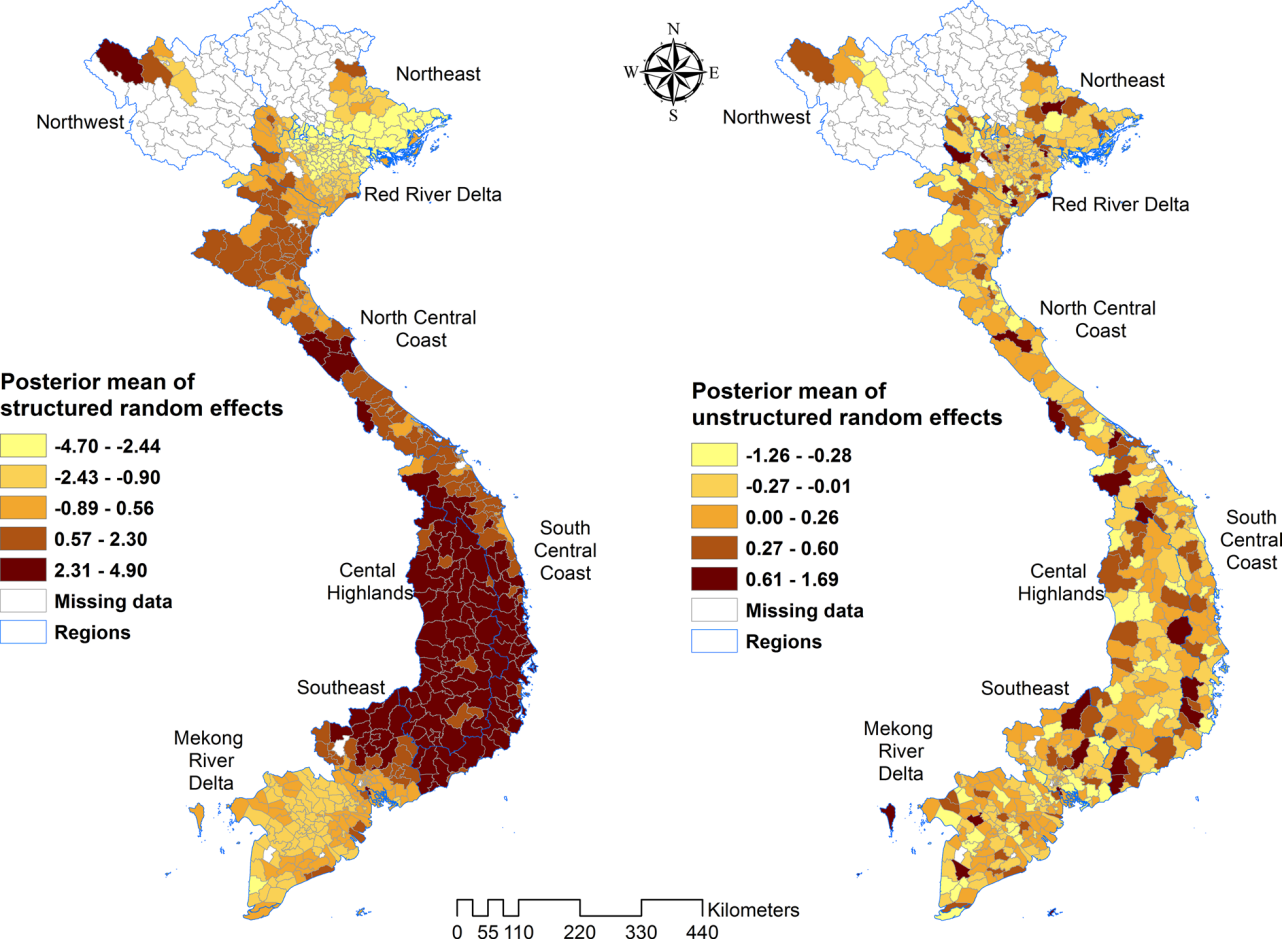
Spatial distribution of the posterior means of structured and unstructured random effects for *Plasmodium vivax* in Model III

Estimation of the spatially auto-correlated random effect (*v*_*i*_) showed higher mean malaria risk of both species in the central and eastern parts of Viet Nam, and lower risk in the southern and north-western areas. As expected, the map of the posterior means of unstructured random effects showed that they were randomly distributed (Figs. 8, 9).

**Fig. 10:**
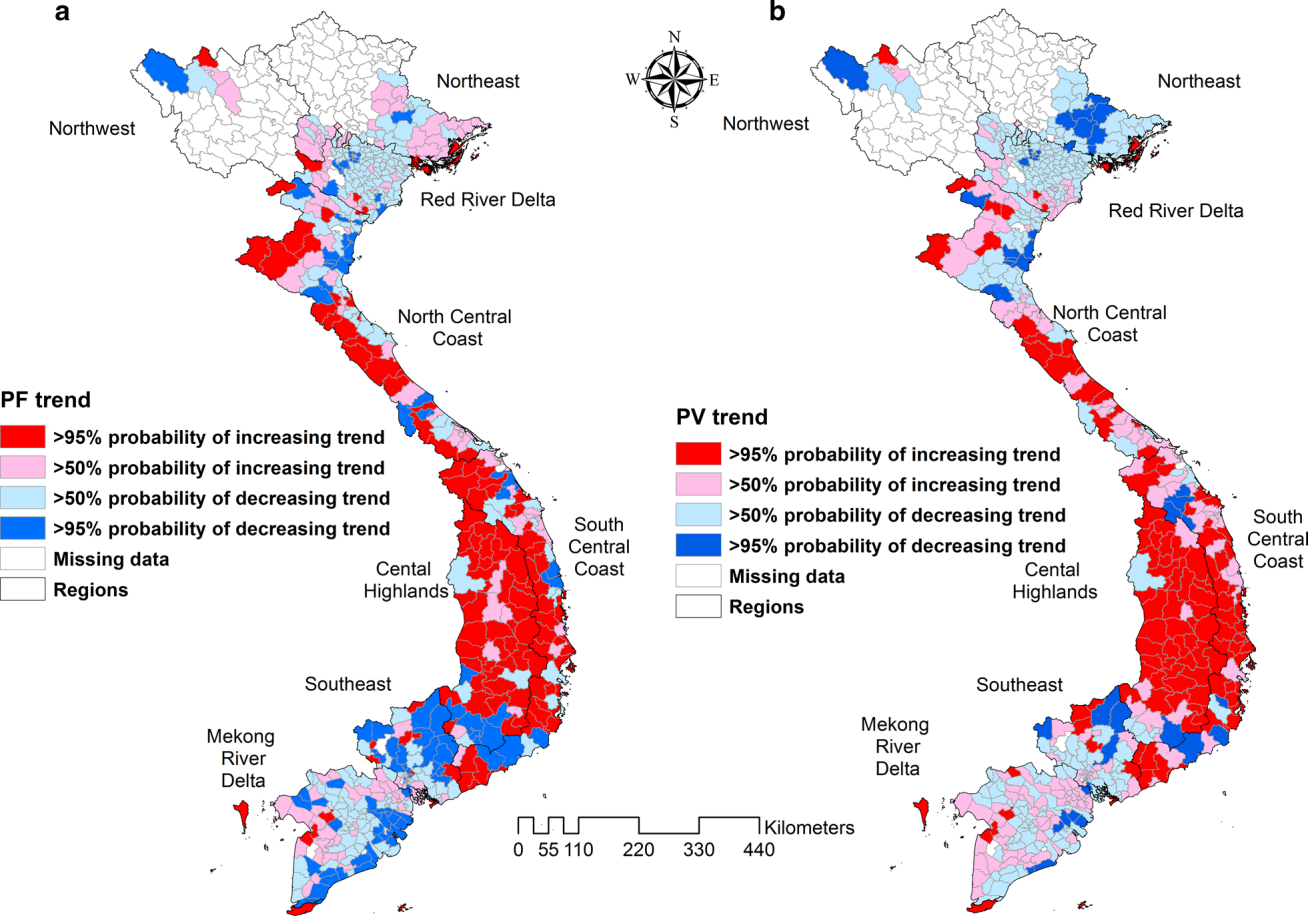
Trend analysis of **a** *Plasmodium falciparum* and **b** *Plasmodium vivax* during the study period 2009–2014

Nationally, Model III showed a significant, positive temporal trend in counts of cases of both types of malaria over the study period. Note, this is after adjusting for the intervention variables, both of which showed a declining trend in the raw data. There was > 95% probability of a higher than national average trend of *P. falciparum* in 134/578 districts, mostly located in the north and south-central coasts and central highlands. Similarly, 127/578 districts had > 95% probability of a higher than national average increasing trend of *P. vivax*, also mostly located in the north and south central coasts and central highlands. For *P. falciparum* and *P. vivax*, 96/578 and 45/578 districts respectively had > 95% probability of a less than national average trend, mostly located in the Red River and Mekong River deltas and the Southeast (Fig. 10).

## Discussion

A Bayesian statistical framework was deployed for disease mapping, with the advantages that both environmental covariates and spatial autocorrelation could be estimated simultaneously and that full posterior distributions are produced, which was used to quantify uncertainties in parameters of interest [44].

Incidence of *P. falciparum* declined during the study period, while *P. vivax* increased from 2009 until 2014, with a slight reduction in incidence in 2015. The reports of resistance of *P. vivax* to chloroquine in Central Viet Nam could have contributed to this increasing trend [45]. Similar findings of increasing *P. vivax* incidence following scale up of control and preventive measures, and a decreasing ratio of *P. falciparum*: *P. vivax* incidence, was reported in Cambodia [46]. Further, it is plausible that *P. vivax* might require different approaches for malaria control and elimination efforts in addition to the currently applied tools.

There was a differential effectiveness of ITN between the two species of *Plasmodium.* A plausible reason could be due to differences in the biting patterns for mosquitoes infected with either *P. falciparum* or *P. vivax* and that there may be greater or lesser vector competence for each species. A study in Papua New Guinea reported that the mosquitoes with *P. vivax* sporozoite showed preferential biting before people retired to beds [47]. The biting pattern, either due to mosquito species or behaviour of malaria-infected mosquitoes, can impact the effectiveness of ITNs and IRS.

Studies have shown that IRS can be more effective in areas with high initial incidence, multiple rounds of spraying and in regions with a combination of *P. falciparum* and *P. vivax* [48]. However, in this study, IRS coverage was not associated with a reduction in malaria risk for either species. The most likely explanation for this is that IRS in Viet Nam has been preferentially targeted at high-risk communities [21, 26] resulting in two-way causation (a type of endogeneity); and suggest that a lack of protection from IRS is a much less likely explanation. A similar result was found for ITN coverage for *P. vivax*. Again, this is likely to be due to the effect of heterogeneous delivery of interventions with preference being given to high-risk communities. Further, it is plausible that houses in Viet Nam, built on elevated columns or stilts with permeable walls, and the biting behaviour of *An. diru*s and *Anopheles minimus*, the primary malaria transmitting mosquitos in Viet Nam, could also play a role in the effectiveness of IRS. The fact that the modelled estimate of national trend in *P. falciparum* risk was positive, when the raw data clearly shows a negative trend, is likely to be due to the adjustment for intervention coverage in the model. Both IRS and ITN coverage at the national level declined during the study period, possibly in response to the declining rates of clinical *P. falciparum* malaria or distribution was focused to malaria transmitting districts only. Further modelling using methods developed in econometrics for dealing with endogeneity should be considered.

The model outputs demonstrated that reported malaria cases predominantly occur in spatial clusters of neighbouring districts. Where spatial clustering is an intrinsic feature of infectious disease data, it is critical to accommodate this phenomenon in statistical analyses to avoid violation of the assumption that observations are independent [8]. Furthermore, the identification of clusters can provide a public health tool to investigate reasons for spatial clustering [35] and to spatially target interventions to high-risk areas. The spatial distribution of both species of malaria was concentrated in the districts in South Central Coasts and South Central Highlands. These were also areas where the temporal trend was significantly higher than the national average. Therefore, strategies should be developed that specifically target these areas to help Viet Nam achieve its malaria elimination goals [27].

High-risk areas included districts that are adjacent to the international borders, where cross-border migration of people from areas of higher malaria endemicity present further challenges to malaria elimination [27]. A study in Cambodia reported significant clusters of high malaria risk located along the Cambodian–Vietnamese border and the Sesan River [49]. Migrant workers, many who live in forested borer regions, have been reported to be at particularly high risk of malaria and may have poor knowledge of malaria and limited access to preventive and therapeutic services [6, 10, 11]. In addition, cross border malaria is likely to contribute to the spread of anti-malarial drug-resistance in Viet Nam [50, 51] through importation of multi-drug resistant malaria from Cambodia [52, 53].

Malaria incidence was strongly associated with maximum temperature. Temperature plays a crucial role in the transmission cycle of malaria parasite and mosquito survival [54]. Studies found that at the temperature of 22 °C, a life cycle of malaria parasite development in mosquito vector is completed at less than 3 weeks [55]. Higher temperatures in Asia have been attributed by strong *El Niño* cycles [56] and multiple studies have investigated the impact of temperature and other climate variables on inter-annual patterns in malaria risk, with potential application to enhanced malaria surveillance [57–59].

One of the major limitations of the study was the use of routine case reports, which suffer from a lack of completeness and representativeness. The passive, routine reporting of malaria cases through the health information system in Viet Nam may underestimate the true number of cases [60]. Whether these factors affect the validity of this analysis depends on the extent to which under-reporting systematically differs between districts. This is currently difficult to determine and should be a focus of future investigation. Secondly, the provinces aiming for malaria elimination are more likely to report every single case of malaria as opposed to those provinces with a large number of cases. Thirdly, the populations of districts were projected from census data and may have led to over or under estimation. Fourthly, unmeasured risk modifiers, such as socio-economic development, living standards, completeness of malaria treatment, localised behavioural patterns and population mobility, and bed net use (as opposed to coverage) are unaccounted for in this study. These variables could also have an effect on the observed spatiotemporal patterns of malaria incidence [61–63].

## Conclusion

Interventions (ITNs distribution) protected the population against *P. falciparum,* while environmental factors (increased temperature) were associated with increased incidence of *P. falciparum* and *P. vivax* during the study period. The factors reviewed were not exhaustive, however the data suggest distribution of resources can be targeted to areas and times of increased malaria transmission. High risk areas of malaria transmission with a high probability of greater than average temporal trends in comparison to the national trend were identified in the central and southern regions of Viet Nam. Targeted distribution of resources in these districts might achieve a greater impact on malaria reduction rather than uniform allocation of resources across the country. The increasing trends of malaria in specific parts of country can also help programme managers in identifying the areas for additional resource allocation for preventive activities. Additionally, changing distribution of the two predominant malaria species in Viet Nam will require different programmatic approaches for control and elimination.

## Additional file

**Additional file 1: Appendix S1.** Climatic variables without lag. **Appendix S2.** Climatic variables with 1 month lag. **Appendix S3.** Climatic variables with 2 months lag. **Appendix S4.** Climatic variables with 3 months lag. **Appendix S5.** Summary of annual distribution of ITNs at district level in Viet Nam from 2009 to 2013. **Appendix S6.** Summary of annual distribution of ITNs of districts included in the study.

## Abbreviations

ACT: artemisinin-based combination therapy
AIC: Akaike’s information criterion
BIC: Bayesian information criterion
CAR: conditional autoregressive
CrI: credible interval
DIC: deviation information criterion
SMR: standardized morbidity ratios
GIS: geographical information systems
GMS: Greater Mekong Sub-region
IRS: indoor residual spraying
ITN: insecticide-treated mosquito nets
LLIN: long-lasting insecticidal nets
MCMC: Markov chain Monte Carlo
NIMPE: National Institute of Malariology, Parasitology, and Entomology
RDT: rapid diagnostic test
WHO: World Health Organization
