# Supplementary material for: Analysis of clinical malaria disease patterns and trends in Vietnam 2009–2015
Source: Malar J. 2018 Sep 17;17:332. doi: 10.1186/s12936-018-2478-z (PMC6142383; doi:10.1186/s12936-018-2478-z)
Supplement: Supplementary file 1 — Additional file 1: Appendix S1. Climatic variables without lag. Appendix S2. Climatic variables with 1 month lag. Appendix S3. Climatic variables with 2 months lag. Appendix S4. Climatic variables with 3 months lag. Appendix S5. Summary of annual distribution of ITNs at district level in Viet Nam from 2009 to 2013. Appendix S6. Summary of annual distribution of ITNs of districts included in the study. [file 12936_2018_2478_MOESM1_ESM.docx]

**Appendix**

**Appendix S1 Climatic variables without lag.**

| **Variables** | **IRR (95% CI)** | **P value** | **AIC** | **BIC** |
| --- | --- | --- | --- | --- |
| Precipitation | 0.999 (0.997, 1.001) | 0.183 | 261.3927 | 268.4515 |
| **Temp max** | **1.003 (1.0028, 1.0032)** | **<0.0001** | **468580.1** | **468597.7** |
| Temp min | 1.0025 (1.0023, 1.0026) | <0.0001 | 468957.4 | 468975.0 |
| Temp mean | 1.0028 (1.0025, 1.0029) | <0.0001 | 468769.4 | 468787.0 |
| **Altitude** | **1.0017 (1.0017, 1.0017)** | **<0.0001** | **431461.3** | **431478.9** |

**Appendix S2 Climatic variables with one month lag.**

| **Variables** | **IRR (95% CI)** | **P value** | **AIC** | **BIC** |
| --- | --- | --- | --- | --- |
| Precipitation | 1.0003 (0.999, 1.002) | 0.714 | 263.2172 | 270.276 |
| **Temp max** | **1.003 (1.0028, 1.0032)** | **<0.0001** | **468615.8** | **468633.4** |
| Temp min | 1.0025 (1.0023, 1.0027) | <0.0001 | 468977.7 | 468995.4 |
| Temp mean | 1.0027 (1.0026, 1.0029) | <0.0001 | 468798.9 | 468798.9 |

**Appendix S3 Climatic variables with two months lag.**

| **Variables** | **IRR (95% CI)** | **P value** | **AIC** | **BIC** |
| --- | --- | --- | --- | --- |
| Precipitation | 1.0012 (0.9996, 1.0029) | 0.133 | 261.2214 | 268.2803 |
| **Temp max** | **1.003 (1.0028, 1.0032)** | **<0.0001** | **468582.5** | **468600.1** |
| Temp min | 1.0025 (1.0024, 1.0027) | <0.0001 | 468956.0 | 468973.6 |
| Temp mean | 1.0028 (1.0026, 1.0029) | <0.0001 | 468773.9 | 468791.6 |

**Appendix S4 Climatic variables with three months lag.**

| **Variables** | **IRR (95% CI)** | **P value** | **AIC** | **BIC** |
| --- | --- | --- | --- | --- |
| Precipitation | 1.002 (0.9999, 1.0031) | 0.064 | 260.1424 | 267.2013 |
| **Temp max** | **1.003 (1.0029, 1.0033)** | **<0.0001** | **468566.5** | **468584.1** |
| Temp min | 1.0025 (1.0024, 1.0027) | <0.0001 | 468960.3 | 468977.9 |
| Temp mean | 1.0028 (1.0025, 1.0029) | <0.0001 | 468768.8 | 468786.4 |

**Appendix S5 Summary of annual distribution of ITNs at district level in Viet Nam from 2009-2013**

| Year | No of district | Annual percent (mean) coverage of ITNs | Min percent of pop covered by ITNs | Max percent of pop covered by ITNs | **Cumulative ITN coverage*** |
| --- | --- | --- | --- | --- | --- |
| **2009** | 357 | 22.6 | 0.001 | 132.1 | 22.6 |
| **2010** | 360 | 22.8 | 0.001 | 121.0 | 45.6 |
| **2011** | 353 | 22.2 | 0.001 | 115.7 | 67.6 |
| **2012** | 351 | 20.1 | 0.001 | 123.1 | 65.1 |
| **2013** | 323 | 20.5 | 0.001 | 101.0 | 62.8 |

* Cumulative ITNs coverage including the coverage of past three years.

pop- population

**Appendix S6 Summary of annual distribution of ITNs of districts included in the study in Viet Nam from 2009-2013 (N=578)**

| Year | No of district | Mean annual coverage of ITNs | Min percent of pop covered by ITNs | Max percent of pop covered by ITNs |
| --- | --- | --- | --- | --- |
| **2009** | 574 | 14.1 | 0 | 132.1 |
| **2010** | 574 | 14.3 | 0 | 121.0 |
| **2011** | 574 | 13.6 | 0 | 115.7 |
| **2012** | 574 | 12.3 | 0 | 123.1 |
| **2013** | 574 | 11.6 | 0 | 101.0 |

pop- population
